# Erlotinib-Related Rash Resolved With Novel Treatment Using Systemic Intravenous Immunoglobulin and Stem Cells

**DOI:** 10.7759/cureus.78078

**Published:** 2025-01-27

**Authors:** Iya A Agha, Erika Malana, Stanley Skopit

**Affiliations:** 1 Dermatology, New York Institute of Technology College of Osteopathic Medicine, New York, USA; 2 Dermatology, Larkin Community Hospital, South Miami, USA

**Keywords:** drug rash, drug reaction, drug reaction treatment, egfr inhibitor-related rash, erlotinib, intravenous immunoglobulin (ivig)

## Abstract

In this report, we describe the case of a 67-year-old Caucasian male with a past medical history of epidermal growth factor receptor (EGFR)-positive non-small-cell lung cancer who presented to the outpatient clinic with a stinging, itchy, red-brown papulopustular rash over his bilateral lower extremities. The patient had been receiving treatment with erlotinib, an EGFR inhibitor, initiated two weeks before the onset of the rash. While not an infrequent reaction to EGFR inhibitors, this patient’s quality of life was significantly affected by the drug reaction. The patient had failed the mainstay of treatment with topical steroids and oral antibiotics and was treated with a combination of intravenous immunoglobulin (IVIG) and stem cells. This led to the complete resolution of the drug eruption which subsequently allowed him to tolerate treatment with erlotinib without recurrence of the drug rash. To our knowledge, we report the first documented case of refractory EGFR inhibitor-induced rash treated with IVIG and stem cells.

## Introduction

The overexpression and dysregulation of epidermal growth factor receptor (EGFR) is not uncommon in solid-organ malignancies, specifically in non-small-cell lung cancer (NSCLC) [1]. EGFR inhibitors such as the monoclonal antibody tyrosine kinase inhibitor, erlotinib, decrease the metastatic potential of EGFR-positive tumors, leading to improved patient outcomes. However, this class of medications is associated with well-documented dermatological adverse events, most pertinently, rash [2]. Proactive management of these cutaneous side effects should be considered given that some studies show over 70% of patients have their EGFR inhibitor treatments paused or stopped altogether secondary to drug reactions [3].

EGFR is expressed ubiquitously in sebaceous glands, epidermis, and hair follicular epithelium where it protects against ultraviolet (UV) radiation, promotes wound healing and normal skin health, and prevents inflammation. Although not completely understood, it is believed that inhibiting EGFR causes occlusion and subsequent rupture of affected follicles leading to the characteristic rash associated with EGFR inhibitors. Furthermore, the imbalance of EGFR inhibition to stimulation leads to an upregulation of genes that promote apoptosis, inflammation, and cell detachment, thereby causing follicles to rupture more readily [4]. Subsequent alteration in permeability of the skin barrier allows for bacterial overgrowth further contributing to the characteristic skin rash.

Before administering the drug, patients should be counseled to utilize sun-protective measures to prevent the progression of the rash and irritation of the already sensitive skin. However, despite these efforts, the rash may worsen to necessitate treatment with topical clindamycin, oral doxycycline, and topical steroids. Should that fail, a decrease in dosage and possible cessation of the EGFR inhibitor may be unfortunate, albeit necessary, considerations given that the medication is likely to provide immense benefit in the patient’s oncologic care [5].

## Case presentation

**Figure 1:**
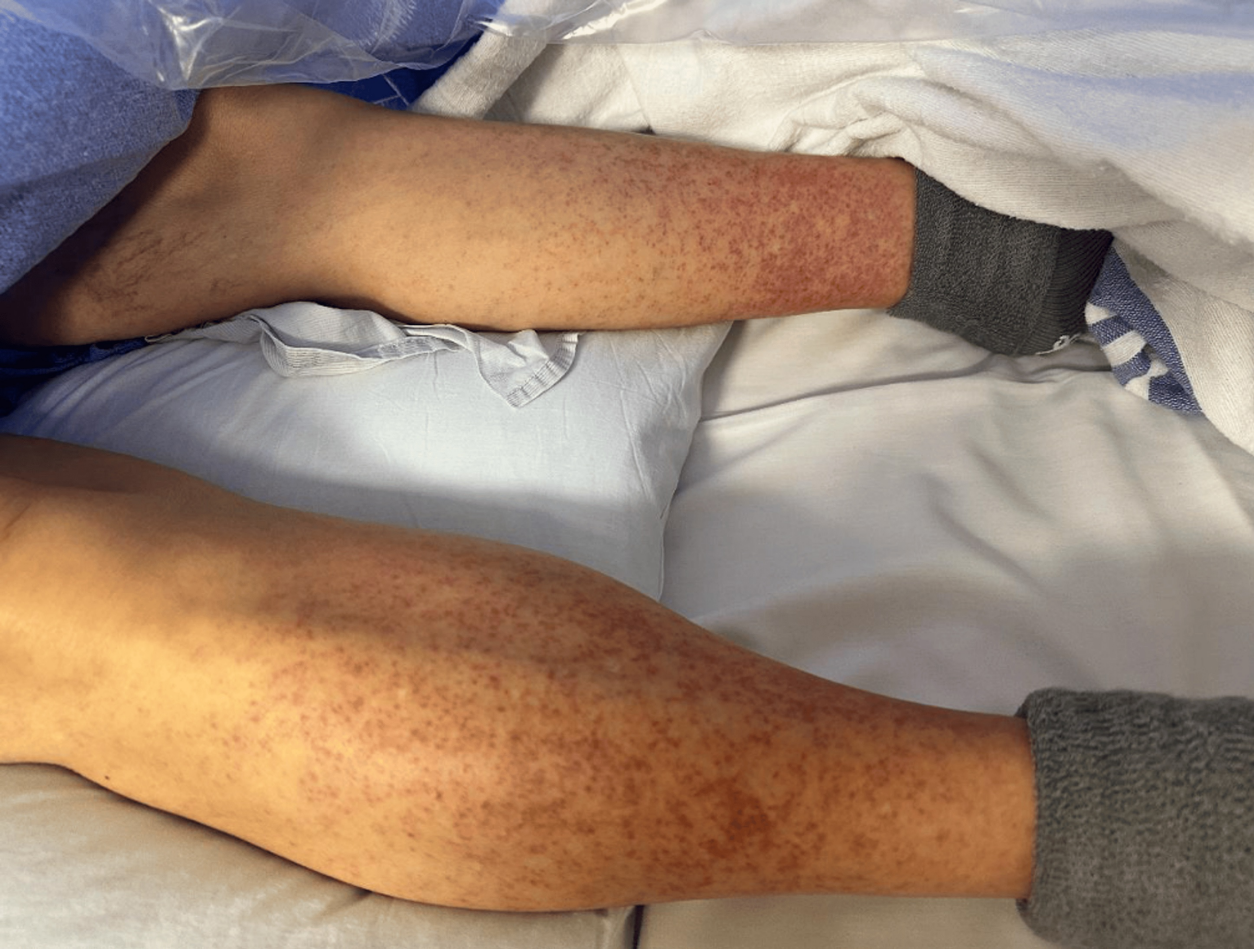
Red to brown papulopustular rash distributed bilaterally on the posterior aspect of the lower extremities. Distribution of the rash was limited to the posterior aspect of the lower extremeties.

A 67-year-old Caucasian male with a past medical history of EGFR-positive NSCLC presented to the outpatient clinic with an erythematous papulopustular rash with underlying erythema on the bilateral lower extremities (Figure 1). The patient endorsed stinging and pruritus and resultant decreased quality of life. The patient first noticed the rash two weeks after starting erlotinib, suggestive of an adverse drug rash within a well-documented timeline [1]. The patient then failed the mainstay of treatment with topical steroids, topical clindamycin, and oral doxycycline. The next step in the escalation of care would have been to lower the dose of or discontinue erlotinib. However, given the oncologic benefit the patient stood to face should he continue erlotinib, shared decision-making was employed to look for other options to salvage his treatment plan.

Low-dose intravenous immunoglobulin (IVIG) and dilute stem cells were initiated to combat the bacterial overgrowth thought to promote the EGFR inhibitor-induced rash while also sparing the patient’s immune system. The patient had 70% improvement within the first 24 hours of therapy, had complete resolution within 96 hours, and has since been able to receive erlotinib treatment without recurrence of any cutaneous findings.

## Discussion

EGFR inhibitors have been used to treat a variety of solid-organ malignancies, including NSCLC, gastrointestinal malignancies, and head and neck cancers [6]. Erlotinib is a medication within this drug class that acts on the transmembrane tyrosine kinase receptor. Cutaneous side effects are common occurrences within this drug class with up to a 90% incidence rate that ranges widely in presentation and severity [7]. Presentations can include a papulopustular rash, as seen in this case, mucositis, nail changes, xerosis, and fissuring of the skin [8]. Furthermore, UV exposure and lighter skin phenotypes may predispose patients to a dose-dependent adverse response to EGFR inhibitors.

EGFR is expressed ubiquitously in keratinocytes of hair follicles, nails, sweat glands, and the epidermis. In addition to plugging and later rupture of affected follicles, there is a disruption of normal keratinocyte function causing leukocytes and neutrophils to incite an inflammatory response. The scalp and face have a higher density of sebaceous glands which makes them more susceptible to the development of the rash. However, areas with a less dense representation of sebaceous glands, such as the legs, can also be affected [9].

Topical corticosteroids and oral doxycycline or minocycline have been given to patients with relative success [5], with refractory cases leading to early cessation of chemotherapy. To our knowledge, we report the first documented case of refractory EGFR inhibitor-induced rash successfully treated with IVIG and stem cells. The patient experienced no adverse effects from said therapy and had resolution of the papulopustular rash without recurrence, allowing him to continue erlotinib treatment.

Further research in the form of randomized clinical trials can be done to elucidate the efficacy of IVIG and stem cell therapy in EGFR inhibitor-induced cutaneous reactions. The pathophysiology of how this therapy contributes to its resolution can be investigated to test the hypothesis of immunological sparing and inform on future treatments for chemotherapy-induced drug rashes. While the exact pathophysiology is unknown, it is theorized that IVIG promotes the immune system to combat the overgrowth of bacteria, while the influx of multipotent stem cells contributes to wound healing. IVIG in conjunction with systemic corticosteroids has successfully treated other drug reactions including drug rash eosinophilia and systemic symptoms (DRESS). In the case of DRESS, IVIG is believed to cause an immunomodulatory effect that allows the patient’s immune system to clear the rash [10]. We theorize that a similar benefit could be applied to the treatment of resistant EGFR inhibitor-related drug reactions.

Oncologists often provide stem cell transplants for patients to replenish healthy cells destroyed by chemotherapy. The use of stem cells for immune reconstitution is continuously being studied [11]. Further studies for their use in conjunction with IVIG can provide an immense benefit for patients with resistant chemotherapy-related drug rashes.

## Conclusions

We present a case of cutaneous toxicity in the form of a papulopustular rash secondary to erlotinib, an EGFR inhibitor used to treat NSCLC. The patient was successfully treated with a combination of IVIG and stem cells. This approach is believed to stimulate the immune system to decrease bacterial colonization and inflammatory cytokines while providing an influx of healthy cells to promote wound healing. With the expanding use of EGFR inhibitors, drug rashes may be a common occurrence in oncology. Proper prevention with sun-protective measures, topical steroids, and oral antibiotics are the mainstay of treatment for these toxicities. However, in patients who are refractory to these treatments, the use of IVIG and stem cells can be explored to allow patients to continue their chemotherapy.
